# Supplementary material for: Identification of BoLA Alleles Associated with BLV Proviral Load in US Beef Cows
Source: Pathogens. 2022 Sep 24;11(10):1093. doi: 10.3390/pathogens11101093 (PMC9610478; doi:10.3390/pathogens11101093)
Supplement: Supplementary file 1 [file pathogens-11-01093-s001.zip › pathogens-1798198-supplementary.pdf]

Supplementary Table S1. Summary Data on Study Animals

COW_ID	BoLA_DRB3_Allele_1	BoLA_DRB3_Allele_2	Genotype	Proviral Load	Age (months)	Farm_Home_State	Breed
1	*018:01	*018:01	1	0	60	MI	.
2	*018:01	*018:01	1	0	84	MI	an
3	*018:01	*018:01	1	0.119	36	MI	an
4	*018:01	*018:01	1	1.028	60	MI	an
5	*018:01	*018:01	1	0.002	48	MI	.
6	*018:01	*018:01	1	0.003	96	MI	an
7	*018:01	*018:01	1	0.031	60	MI	an/sm
8	*018:01	*018:01	1	0.067	36	MI	an
9	*018:01	*018:01	1	0.186	168	MI	an
10	*018:01	*018:01	1	0.268	48	MI	an
11	*018:01	*018:01	1	0.282	24	IA	an
12	*018:01	*018:01	1	0.304	50	MI	an
13	*018:01	*018:01	1	0.427	60	MI	an
14	*018:01	*018:01	1	0.558	60	MI	an
15	*018:01	*018:01	1	0.761	108	MI	an
16	*018:01	*018:01	1	0.825	36	MI	an
17	*018:01	*018:01	1	0.94	84	MI	an
18	*018:01	*018:01	1	1.073	36	MI	an
19	*018:01	*018:01	1	1.083	72	MI	an
20	*018:01	*018:01	1	1.097	108	MI	an
21	*018:01	*018:01	1	1.191	72	MI	.
22	*018:01	*018:01	1	1.214	48	IA	an
23	*018:01	*018:01	1	1.226	60	MI	an
24	*018:01	*018:01	1	1.418	108	MI	an
25	*018:01	*018:01	1	1.716	24	MI	an/sm
26	*026:01	*026:01	2	0	84	MI	an
27	*026:01	*026:01	2	0.202	60	MI	an

28	*026:01	*026:01	2	1.38	84	MI	an/sm
29	*026:01	*026:01	2	1.609	120	MI	an/ho
30	*026:01	*026:01	2	0.152	108	MI	an
31	*026:01	*026:01	2	0.16	60	IA	an
32	*026:01	*026:01	2	0.185	73	MI	an
33	*026:01	*026:01	2	0.203	156	MI	an
34	*026:01	*026:01	2	0.255	24	MI	an
35	*026:01	*026:01	2	0.363	72	MI	an/sm
36	*026:01	*026:01	2	0.543	36	MI	an
37	*026:01	*026:01	2	0.662	48	MI	an
38	*026:01	*026:01	2	0.674	36	MI	an
39	*026:01	*026:01	2	0.841	36	MI	an
40	*026:01	*026:01	2	1.078	24	MI	an
41	*026:01	*026:01	2	1.082	108	MI	an
42	*026:01	*026:01	2	1.254	97	MI	an
43	*026:01	*026:01	2	1.287	108	MI	an
44	*026:01	*026:01	2	1.341	51	MI	an
45	*026:01	*026:01	2	1.598	108	MI	an
46	*033:01	*018:01	3	0	60	MI	an
47	*033:01	*018:01	3	0	84	IA	an
48	*033:01	*018:01	3	0	95	MI	an
49	*033:01	*018:01	3	0.013	24	MI	an
50	*033:01	*018:01	3	1.106	48	MI	an
51	*002:01	*002:01	4	0	72	MI	an
52	*002:01	*002:01	4	0	24	MI	.
53	*002:01	*002:01	4	0	60	MI	an
54	*002:01	*002:01	4	0	84	MI	an
55	*002:01	*002:01	4	0	156	MI	an
56	*002:01	*002:01	4	0	48	MI	an
57	*002:01	*002:01	4	0	60	MI	an/sm

58	*002:01	*002:01	4	0	48	MI	an
59	*002:01	*002:01	4	0	86	MI	an
60	*002:01	*002:01	4	0.106	72	MI	an/sm
61	*002:01	*002:01	4	0.113	60	MI	an/sm
62	*002:01	*002:01	4	0.224	36	MI	an
63	*002:01	*002:01	4	0	72	MI	an
64	*002:01	*002:01	4	0.004	36	MI	.
65	*002:01	*002:01	4	0.011	84	MI	an
66	*002:01	*002:01	4	0.051	60	MI	an
67	*002:01	*002:01	4	0.078	96	MI	an
68	*002:01	*002:01	4	0.092	60	MI	an
69	*002:01	*002:01	4	0.133	36	MI	an
70	*002:01	*002:01	4	0.171	48	IA	an
71	*002:01	*002:01	4	0.174	48	IA	an
72	*002:01	*002:01	4	0.219	60	MI	an
73	*002:01	*002:01	4	0.22	60	IA	an
74	*002:01	*002:01	4	0.235	72	MI	an/sm
75	*002:01	*002:01	4	0.576	108	MI	an/sm
76	*002:01	*002:01	4	0.882	36	MI	an
77	*002:01	*018:01	5	0	72	MI	an
78	*002:01	*018:01	5	0	48	MI	an
79	*002:01	*018:01	5	0	36	MI	an
80	*002:01	*018:01	5	0	108	MI	an
81	*002:01	*018:01	5	0	60	IA	an
82	*002:01	*018:01	5	0	72	MI	an
83	*002:01	*018:01	5	0	48	MI	an
84	*002:01	*018:01	5	1.283	108	MI	an
85	*002:01	*018:01	5	0.003	48	MI	.
86	*002:01	*018:01	5	0.044	62	MI	an
87	*002:01	*018:01	5	0.091	83	MI	an

88	*002:01	*018:01	5	0.176	108	MI	an
89	*002:01	*018:01	5	0.253	168	MI	an
90	*002:01	*018:01	5	0.496	24	MI	an
91	*002:01	*018:01	5	0.57	144	MI	an
92	*002:01	*018:01	5	0.901	84	MI	an
93	*002:01	*018:01	5	0.996	48	MI	an
94	*018:01	*002:01	5	1.088	35	MI	an
95	*018:01	*002:01	5	1.242	60	MI	an
96	*002:01	*018:01	5	1.31	96	MI	an
97	*002:01	*018:01	5	1.315	60	MI	an
98	*002:01	*032:01	6	0	24	MI	an
99	*033:01	*033:01	7	0	84	MI	an
100	*002:01	*033:01	8	0	72	MI	an
101	*033:01	*002:01	8	0	48	IA	an
102	*002:01	*016:01	9	0	60	MI	an
103	*016:01	*002:01	9	0.028	132	MI	sm
104	*008:01	*026:01	10	0	36	MI	an
105	*026:01	*002:01	11	0	48	IA	an
106	*026:01	*002:01	11	0	48	IA	an
107	*002:01	*026:01	11	1.704	96	MI	an/sm
108	*026:01	*002:01	11	0.001	24	MI	an
109	*026:01	*002:01	11	0.04	96	MI	an
110	*026:01	*002:01	11	0.603	36	MI	an
111	*026:01	*002:01	11	0.917	84	MI	an
112	*002:01	*026:01	11	1.174	120	MI	an
113	*026:01	*002:01	11	1.603	84	MI	an/sm
114	*002:01	*001:01	12	0	84	IA	an
115	*002:01	*001:01	12	0.201	24	MI	an
116	*001:01	*002:01	12	0.295	73	MI	an
118	*002:01	*010:01	14	0	96	MI	an/sm

119	*033:01	*037:01	15	0	48	MI	an
120	*018:01	*026:01	16	0.046	60	MI	an/sm
121	*026:01	*018:01	16	1.012	36	MI	an
122	*026:01	*018:01	16	1.304	48	MI	an/sm
123	*026:01	*018:01	16	0.076	60	MI	an
124	*026:01	*018:01	16	0.093	24	MI	an
125	*026:01	*018:01	16	0.271	36	MI	an
126	*026:01	*018:01	16	0.304	24	MI	an
127	*026:01	*018:01	16	0.401	120	MI	an
128	*026:01	*018:01	16	0.452	72	MI	an
129	*026:01	*018:01	16	1.049	36	MI	an
130	*026:01	*018:01	16	1.51	24	MI	an
131	*026:01	*018:01	16	1.681	72	MI	an
132	*026:01	*018:01	16	2.008	72	MI	an
133	*026:01	*018:01	16	2.543	60	IA	an
134	*048:02	*048:02	17	0.1	84	MI	an
135	*048:02	*048:02	17	0.65	24	MI	.
136	*007:01	*007:01	18	0.21	84	MI	an/sm
137	*007:01	*007:01	18	0.376	72	MI	an
138	*007:01	*007:01	18	0.047	96	MI	an
139	*016:01	*016:01	19	0.387	60	MI	an
140	*008:01	*018:01	20	0.476	72	MI	an/sm
141	*008:01	*018:01	20	0.827	36	MI	an
142	*009:02	*009:02	21	1.124	36	MI	an/sm
143	*009:01	*009:01	23	0.009	134	MI	an
144	*008:01	*008:01	24	0.796	60	MI	an/sm
145	*008:01	*008:01	24	1.912	108	MI	.
146	*015:01	*015:01	25	0.053	60	IA	an
147	*011:01	*026:01	26	0.066	48	IA	an
148	*032:01	*018:01	27	0.133	60	MI	an

149	*018:01	*032:01	27	0.43	24	MI	an
150	*018:01	*032:01	27	0.862	48	IA	an
151	*018:01	*032:01	27	1.79	108	MI	.
152	*007:01	*026:01	28	0.237	144	MI	an
153	*032:01	*001:01	29	0.467	108	MI	an
154	*002:01	*009:01	30	0.793	24	MI	an/sm
155	*018:01	*015:01	31	0.834	72	MI	.
156	*006:01	*006:01	32	1.001	36	MI	an
157	*018:01	*001:01	33	2.086	60	IA	an

*indicates allele at the BoLA-DRB3 gene locus.

Supplementary Figure S1.

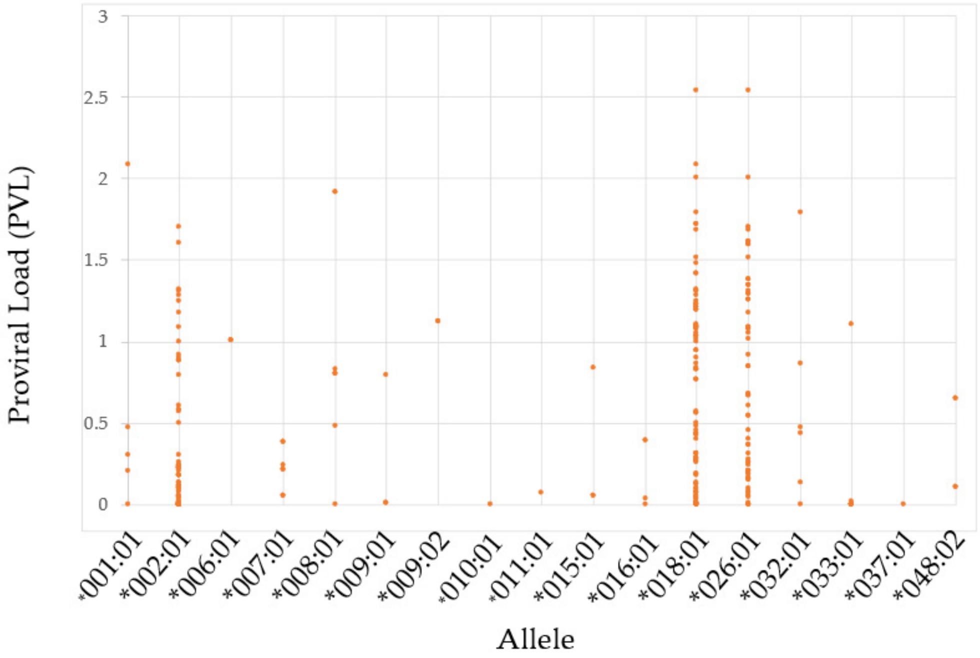

***indicates allele at the BoLA-DRB3 gene locus.**

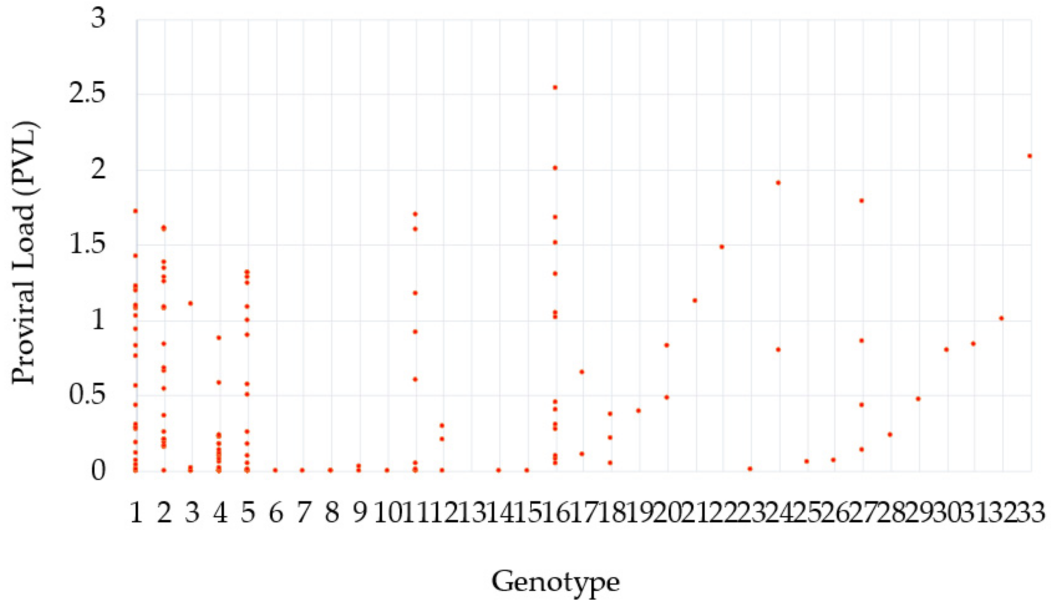

Supplementary Figure S1. Proviral Loads of Study Population based upon Alleles and Genotypes. Red dots indicate one animal. Genotypes are organized as described in Supplementary Table 2.

Supplementary Table S2. Genotypes and Respective Frequencies within Study Population

Genotype	BoLA_DRB3_Allele_1	BoLA_DRB3_Allele_2	Individual Count	Genotypic Frequency
1	*018:01	*018:01	25	0.1592
2	*026:01	*026:01	20	0.1274
3	*018:01	*033:01	5	0.0318
4	*002:01	*002:01	26	0.1656
5	*002:01	*018:01	21	0.1338
6	*002:01	*032:01	1	0.0064
7	*033:01	*033:01	1	0.0064
8	*002:01	*033:01	2	0.0127
9	*002:01	*016:01	2	0.0127
10	*008:01	*026:01	1	0.0064
11	*002:01	*026:01	9	0.0573
12	*001:01	*002:01	3	0.0191
13	*037:01	*037:01	1	0.0064
14	*002:01	*010:01	1	0.0064
15	*033:01	*037:01	1	0.0064
16	*018:01	*026:01	14	0.0892
17	*048:02	*048:02	2	0.0127
18	*007:01	*007:01	3	0.0191

19	*016:01	*016:01	1	0.0064
20	*008:01	*018:01	2	0.0127
21	*009:02	*009:02	1	0.0064
23	*009:01	*009:01	1	0.0064
24	*008:01	*008:01	2	0.0127
25	*015:01	*015:01	1	0.0064
26	*011:01	*026:01	1	0.0064
27	*018:01	*032:01	4	0.0255
28	*007:01	*026:01	1	0.0064
29	*001:01	*032:01	1	0.0064
30	*002:01	*009:01	1	0.0064
31	*015:01	*018:01	1	0.0064
32	*006:01	*006:01	1	0.0064
33	*001:01	*018:01	1	0.0064
	TOTAL		157	1

*indicates allele at the BoLA-DRB3 gene locus.
